# Differential contribution of elastin and fibrillin-1 to the cardiovascular phenotype of a double heterozygous Marfan and Williams-Beuren syndrome mouse model

**DOI:** 10.1016/j.gendis.2026.102268

**Published:** 2026-06-01

**Authors:** Isaac Rodríguez-Rovira, Lize Sels, Jana Ruíz-Castro, Alba Aizpuru-Gómez, Ana Paula Dantas, Victoria Campuzano, Gustavo Egea

**Affiliations:** a Department of Biomedical Sciences, School of Medicine and Health Sciences, University of Barcelona, Barcelona 08036, Spain; b Institute of Biomedical Research August Pi Sunyer (IDIBAPS), Barcelona 08036, Spain; c Institut Clínic Cardiovascular (ICCV), Hospital Clínic, Barcelona 08036, Spain; d Rare Diseases Biomedical Research Network Center (CIBERER), ISCIII, Madrid 28029, Spain; e Center of Medical Genetics, University of Antwerpen, Edegem 2659, Belgium

Marfan syndrome (MFS; OMIM 154700) and Williams-Beuren syndrome (WBS, OMIM 194050) are genetic diseases caused respectively by mutations in the fibrillin-1 gene (*FBN1*) and a microdeletion including hemizygous loss of the elastin gene (*ELN*). Their respective vascular manifestations are opposed, thoracic aortic aneurysm in MFS and supravalvular aortic stenosis in WBS.^1^ To investigate the interdependence of both constituents of elastic fibres in their cardiovascular pathology, we generated a double heterozygous mouse model (CDMFS) by crossing MFS female (*Fbn1*^*C1041G/+*^) with (CD)-WBS like male.^2^^,^^3^ CDMFS mice developed the aortic root aneurysm, which progressed indistinguishably from that in MFS mice. CDMFS aortic wall showed a thicker tunica media and thinner elastic fibres consistent with CD mice. The reduction in vascular smooth muscle cell density in MFS aorta was not observed in CDMFS aorta. CDMFS mice developed the characteristic hypertension observed in CD animals, which does not occur in MFS. Cardiac ejection fraction was equally reduced in MFS, CD, and CDMFS mice. Cardiac redox stress and mitochondrial function increased and reduced respectively only in CD mice, both being normalised in CDMFS animals. Metalloproteinase-2 protein levels increased only in MFS hearts being normalized in CDMFS hearts. In conclusion, CDMFS mice mostly exhibited the characteristic phenotypic manifestations of each syndrome, along with the pathological outcomes associated with each elastic fibre component, consistent with their respective associated diseases.

Segregation of double heterozygous CDMFS mice when crossing single heterozygous MFS female with single heterozygous CD male followed the expected Mendelian ratios (χ^2^_(3)_ = 7.01, *p* = 0.07), although the number of CDMFS animals was half that of wild-type (WT) animals (Table S1). Double heterozygote CDMFS mice followed a growth line similar to the simple heterozygote CD mice (Fig. S1). A significant effect of genotype was observed for body weight both in males (F_(3,44)_ = 53.28, *p* < 0.0001) and females (F_(3,47)_ = 24.67, *p* < 0.0001). Due to somatic growth, we observed a significant effect over time for males (F_(1,847,62,17)_ = 106.4, *p* < 0.0001) and females (F_(2,142,74,27)_ = 98.19, *p* < 0.0001). However, there were no changes in the interaction between both factors (genotype and time) for males (F_(9,101)_ = 0.84, *p* = 0.57) or females (F_(9,104)_ = 1.09, *p* = 0.37), which indicates that somatic growth progresses similarly. Therefore, body weight results indicate that the CDMFS phenotype seems to be exclusively determined by the CD phenotype, regardless of sex (Table S1).

Using ultrasonography, we next evaluated aortic root diameter (AR). Double heterozygous CDMFS animals showed AR dilatation (Fig. 1A, purple line) that was indistinguishable from that in MFS mice. In CD mice, the results of intrinsic stenosis showed a reduced AR diameter, evident as early as in two-month-old animals (Fig. 1A, blue line). The AR progression over time (Fig. 1A) indicates a significant effect of both genotype (F_(3,125)_ = 74.88, *p* < 0.0001) and time (F_(1,125)_ = 80.24, *p* < 0.0001), but no interaction between these factors (F_(3,125)_ = 0.67, *p* = 0.56), indicating that the temporal progression of AR growth is similar across all genotypes. Therefore, the results indicate that in CDMFS mice the characteristic MFS-associated AR dilatation predominates over the CD/WBS-associated aortic stenosis (Table S2).

**Figure 1:**
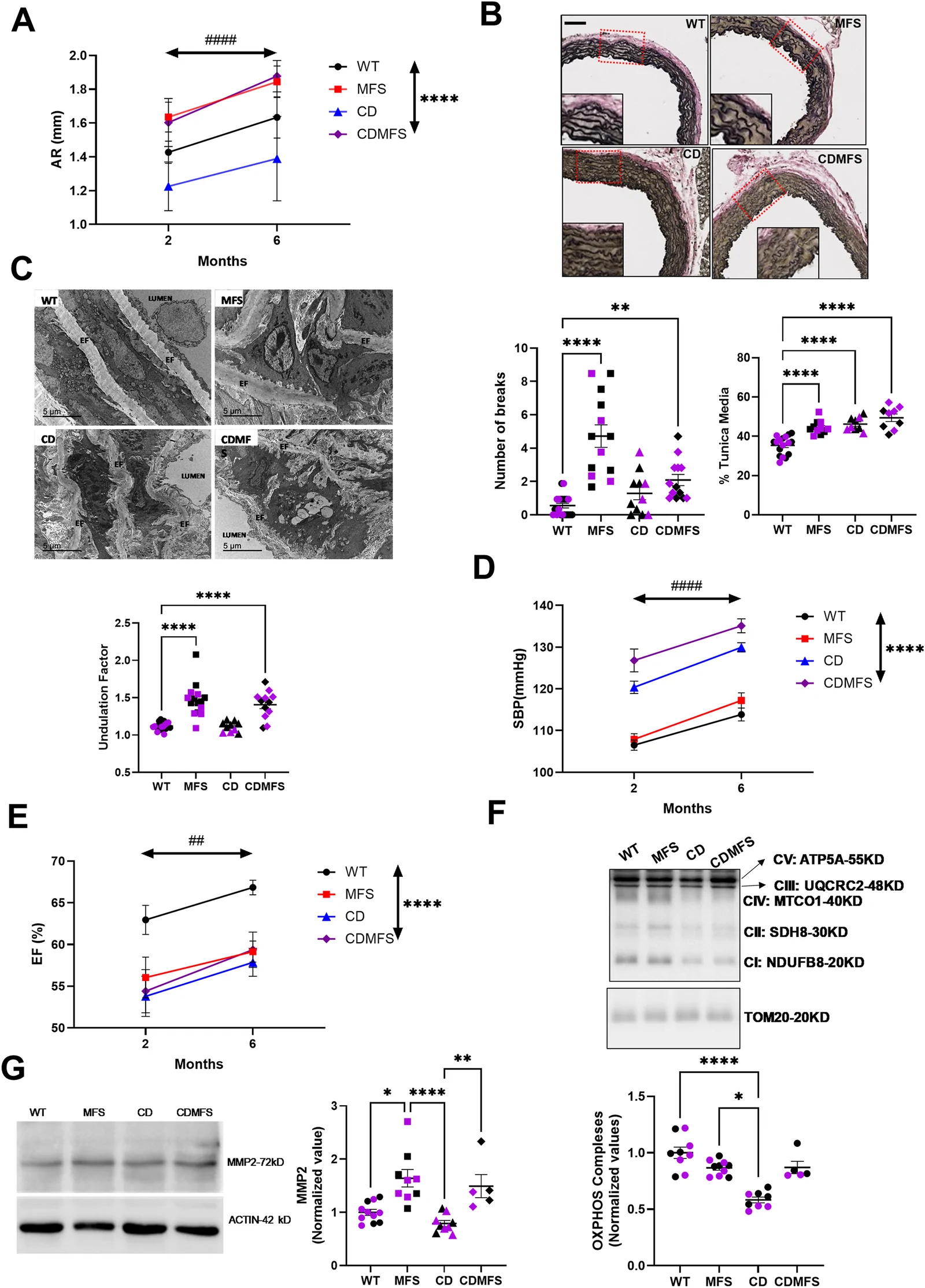
Differential contribution of Elastin and Fibrillin-1 to cardiovascular outcomes. **(A)** Progression of aortic root (AR) diameter. An increase is seen over time (F_(1,125)_ = 80.24, *p* < 0.0001), similar in all genotypes (interaction, F_(3,125)_ = 0.125, *p =* 0.5667). Statistical significance was calculated using two-way mixed effect analysis. ∗Genotype effect; ^#^time effect. ∗∗∗∗^, ####^*p* < 0.0001. **(B)** Elastic laminae organization. Representative pictures of ascending aorta slides stained with Van Gieson's procedure. Scale bar, 100 μm. Insets show magnified selected areas of the indicated aortic wall (red dotted rectangles). Left below: histogram of large elastic fibre breaks. Right below: Histogram showing the percentage of the total area occupied by the tunica media. Statistical significance was calculated by the Kruskal–Wallis test followed by Dunn's multiple comparisons test. ∗∗*p* < 0.01; ∗∗∗∗*p* < 0.0001. **(C)** Electron microscopy of the aortic media. Ultrathin sections of WT, MFS, CD, and CDMFS aortae display the morphology of aortic media vascular smooth muscle cells (VSMC) and elastic fibres. Below, quantitative assessment of the elastic laminae undulation factor. **(D)** Systolic blood pressure (SBP) progression over time. Statistical significance was calculated by two-way mixed effect analysis. ∗Genotype effect; ^#^time effect. ∗∗∗∗^, ####^*p* < 0.0001. **(E)** Ejection fraction progression over time. Statistical significance was calculated by two-way Mixed effect analysis followed by a post-hoc Dunnett's multiple comparisons test. ∗Genotype effect; ^#^time effect, ^##^*p* < 0.01; ∗∗∗∗*p* < 0.0001. **(F)** Representative Western blot for OXPHOS protein complexes in cardiomyocytes; below, quantitative analysis of OXPHOS complex proteins normalised against the mitochondrial protein housekeeping TOM20. (G) Left ventricle MMP-2 levels. Representative Western blot for MMP-2 protein expression levels with corresponding quantitative analysis normalized to G-actin and expressed relative to the mean of WT littermates. Statistical significance in F and G was calculated using the Kruskal–Wallis test followed by Dunn's multiple comparisons test. ∗*p* < 0.05; ∗∗*p* < 0.01; ∗∗∗*p* < 0.001. For all quantitative panels, data are the mean ± SEM. Circles, WT; Squares, MFS; Triangles, CD; Diamonds, CDMFS; Black, males; Purple, females.

Thereafter, we measured several histological aortic parameters (Fig. S2A for representative images). The total aortic area in CDMFS aortae was reduced compared with WT littermates, being indistinguishable from that of CD aortae and in contrast to MFS mice (Fig. S2B). Elastic laminae organization (see representative images in Fig. 1B) was altered compared with WT littermates although to a lesser extent than in MFS aortae (*p* ≤ 0.0001); however, this was not the case for CD aortae (*p* = 0.39) (Fig. 1B, number of breaks). Strikingly, the percentage of the media with respect to the total aortic area was increased in all mouse groups (Fig. 1B, % tunica media). The ultrastructural analysis of the aortae showed reduced elastic fibre amplitude and augmented undulation (enlarged insets in Fig. 1B and ultrastructural images in Fig. 1C). The apparent differences between the echocardiographic and histological analyses could be determined by different pressurization of the aortas and would be in accordance with previously reported mechanical studies (see supplemental Discussion). Vascular smooth muscle cell (VSMC) density in the tunica media showed the characteristic atrophy and hyperplasia corresponding to MFS and CD mice respectively, but CDMFS media showed values highly similar to those observed in WT aortae (*p* = 0.65) (Fig. S2C and Table S3). The characteristic continuous interaction of VSMCs with their adjacent elastic fibres was altered in MFS and severely accentuated in CDMFS aortae (ultrastructural images in Fig. 1C). VSMCs showed few and occasional interactions with neighbouring elastic fibres, exhibiting a comb teeth-like phenotype. Moreover, numerous collagen fibres can be seen in the free spaces that remain between these occasional interactions. This proliferation of collagen fibres suggests a compensatory fibrotic-like response against elastic fibre disarrays.

Unlike MFS patients and mice, WBS patients and CD mice have elevated systolic blood pressure (SBP). The progression of the SBP over time (Fig. 1D) showed that CDMFS mice already present increased SBP at two months of age (*p* = 0.001) and accentuated at six months (*p* < 0.0001) (Fig. 1D, purple line). Likewise, a time effect is observed (F_(1,61)_ = 67.24, *p* < 0.0001) without a genotype–time interaction (F_(3,61)_ = 0.23, *p* = 0.87) because the increase occurs equally across all genotypes with age (Table S4). As a functional indicator of cardiac function, we also evaluated the ejection fraction (EF) in all experimental mouse groups over the age (Fig. 1E). All mutant groups showed a reduced EF compared with WT littermates. We observed a significant effect of genotype (F_(3,113)_ = 17.64, *p* < 0.0001) in agreement with the data analysed at different time points. There was also a significant effect of time (F_(1,113)_ = 12.4, *p* = 0.0006; Table S5). The significant increase observed in EF over time could be attributable to intrinsic physiological changes associated with age.

Mitochondrial alterations and redox stress contribute to cardiovascular damage in genetic-origin aortic diseases. To this aim, we first evaluated protein expression levels of the mitochondrial oxidative phosphorylation system (OXPHOS) complexes.^4^ Only CD cardiomyocytes had reduced protein levels of all complexes (F_(3,27)_ = 26.15, *p* < 0.0001, effect of genotype) (Fig. 1F), whereas both MFS and CDMFS showed a reduced trend that did not reach statistical significance. At the ultrastructural level (Fig. S3), CD mitochondria were aligned and joined together at a single point. This peculiar morphology was also seen in CDMFS and MFS cardiomyocytes, though not as pronounced as in CD. We next evaluated cardiac redox stress levels by dihydroethidium (DHE) fluorescent staining of the left ventricle of six-month-old mice from the experimental groups (Fig. S4). Double heterozygous CDMFS mice showed no differences with respect to their WT siblings (*p* = 0.99). However, CD mice showed a significant increase (Table S6). Finally, postulated redox stress-associated changes in metalloproteinase 2 (MMP2) protein expression were suggested as a potential biomarker of cardiac damage in myocardial infarction and hypertension-associated heart diseases.^5^ The cardiac MMP2/actin ratio was largely increased in MFS (*p* = 0.0006), but not in CD (*p* = 0.40) or CDMFS (Fig. 1G and Table S7), which suggests that increased MMP2 expression level in cardiomyocytes is primarily determined by fibrilin-1.

Cardiovascular disorders in the aorta show a high level of independence with respect to the aortic dilatation but complementarity concerning the organization of the aortic wall and its disarray. At the cardiac level, elastin and fibrillin-1 also have a complementary impact regarding mitochondrial dysfunction (OXPHOS) and MMP2 expression, respectively. Finally, SBP and cardiopathy results in CDMFS mice indicate that both are not necessarily linked, as often presumed, while *Fbn1* haploinsufficiency could participate, somehow in enhancing the characteristic intrinsic hypertension of the CD model. Therefore, the distinct phenotypes and the lack of exacerbation of the single phenotypes in the CDMFS mouse indicate that both syndromes encompass more than an elastin fibre defect, which should divert the focus to other contributors to these diseases.

In summary, the results in the doble heterozygous CDMFS mouse model provide novel insights into the interdependence of fibrillin-1 and elastin proteins in diversed cardiovascular manifestations. An extended discussion and a comparative table summarizing the present findings from mouse models and patients with MFS and WBS (Table S8) are included in supplemental material.

## CRediT authorship contribution statement

**Isaac Rodríguez-Rovira:** Writing – review & editing, Writing – original draft, Supervision, Methodology, Investigation, Formal analysis, Data curation. **Lize Sels:** Writing – review & editing, Investigation, Data curation. **Jana Ruíz-Castro:** Writing – review & editing, Investigation, Data curation. **Alba Aizpuru-Gómez:** Writing – review & editing, Investigation, Formal analysis, Data curation. **Ana Paula Dantas:** Writing – review & editing, Supervision, Data curation. **Victoria Campuzano:** Writing – review & editing, Writing – original draft, Supervision, Resources, Project administration, Formal analysis, Data curation, Conceptualization. **Gustavo Egea:** Writing – review & editing, Writing – original draft, Supervision, Funding acquisition, Formal analysis, Conceptualization.

## Ethics declaration

All animal procedures were conducted following ARRIVE (Animals in Research: Reporting *In Vivo* Experiments) guidelines [1] and standard ethical guidelines (European Union Directive 2010/63/EU), and were approved by the local ethics committee (Animal Experimentation Ethics Committee-University of Barcelona, CEEA-UB) and the Government of Catalonia (No. 106/24).

## Data availability

Data is available upon reasonable requests of corresponding authors.

## Funding

This work was supported by grants from the Ministerio de Ciencia e Innovación [No. PID2021-124799OB-I00 (AEI/MINEICO/FEDER, UE) to VC, No. PID2023-146296OB-I00 to GE]; the Association Autour des Williams”/Federation Williams France to VC; Fundació Marató de TV3 to G.E., and Generalitat de Catalunya (No. 2021 SGR 00029).

## Conflict of interests

The authors declare no conflict of interests.
