# Supplementary material for: Differential contribution of elastin and fibrillin-1 to the cardiovascular phenotype of a double heterozygous Marfan and Williams-Beuren syndrome mouse model
Source: Genes Dis. 2026 Jun 1;14(2):102268. doi: 10.1016/j.gendis.2026.102268 (PMC13634561; doi:10.1016/j.gendis.2026.102268)
Supplement: Supplementary file 1 [file mmc1.docx]

**SUPPLEMENTARY INFORMATION**

**DIFFERENTIAL CONTRIBUTION OF ELASTIN AND FIBRILLIN-1 TO THE CARDIOVASCULAR PHENOTYPE OF A DOUBLE HETEROZYGOUS MARFAN AND WILLIAMS-BEUREN SYNDROME MOUSE MODEL**

Isaac Rodríguez-Rovira^1^, Lize Sels^1^*, Jana Ruíz-Castro^1^, Alba Aizpuru-Gómez^1^, Ana Paula Dantas^1, 2,3^, Victoria Campuzano^1,2,4^**, and Gustavo Egea^1,2,5^**

**Current address; **co-corresponding authors*

^1^Department of Biomedical Sciences, School of Medicine and Health Sciences, University of Barcelona, 08036 Barcelona (Spain).

^2^ Institute of Biomedical Research August Pi Sunyer (IDIBAPS), 08036 Barcelona, Spain.

^3^ Institut Clínic Cardiovascular (ICCV), Hospital Clínic, 08036 Barcelona, Spain.

^4^ Rare Diseases Biomedical Research Network Center (CIBERER), ISCIII, 28029 Madrid (Spain).

^5^ Center of Medical Genetics, University of Antwerpen, 2659 Edegem (Belgium).

*Present address: Department of Translational Neurosciences, Faculty of Medicine and Health Sciences, and Vaccine and Infectious Disease Institute (Vaxinfectio), University of Antwerp, Antwerp (Belgium).

****** Corresponding authors:

Victoria Campuzano: [vcampuzanou@ub.edu](mailto:vcampuzanou@ub.edu)

Gustavo Egea: [gegea@ub.edu](mailto:gegea@ub.edu)

**SUPPLEMENTAL MATERIAL**

*Ethics*

All animal procedures were conducted following ARRIVE (Animals in Research: Reporting In Vivo Experiments) guidelines [1] and standard ethical guidelines (European Union Directive 2010/63/EU) and were approved by the local ethics committee (Animal Experimentation Ethics Committee-University of Barcelona, CEEA-UB) and the Government of Catalonia with the number 106/24.

*Animal models*

Mice harbouring a fibrillin-1 mutation (*Fbn1^C1041G/+^*, referred to as C1041G and previously as C1039G/+; hereafter MFS mice) were obtained from The Jackson Laboratory (B6.129-Fbn1tm1Hcd/J; Strain #012885). Mice with hemizygous deletion of the 7q11.23 region of chromosome 7 (CD mice) were obtained as previously described [2]. All mice were maintained in a C57BL/6J background (backcrossed for nine generations). We obtained the CD-Marfan mice colony (CDMFS) from the breeding of MFS females with CD males. The opposite breeding of MFS males with CD females was discarded because CD females are known to give birth to poor litters, which, moreover, they neglect [2]. The experimental groups were wild-type (WT), MFS (Marfan syndrome), CD (WBS), and CDMFS (double heterozygous CD/MFS) mice. Mice were housed in controlled environmental conditions (21 ± 1°C temperature and 55 ± 10% humidity), and food and water were available *ad libitum*. All experiments were performed during the light phase of a 12-h light/dark cycle (light on at 8 am; light off at 8 pm). Genomic DNA was extracted from mouse ear punch to determine the genotype of each mouse using PCR, as previously described [3].

*Blood pressure measurement*

Systolic blood pressure (SBP) was measured in conscious mice using a tail-cuff system plethysmometer (Non-Invasive Blood Pressure System, PanLab, Barcelona, Spain) while the mice were placed in a restrainer tube, which was cleaned after each use. Following habituation to the restrainer, measurements were taken on three separate occasions and averaged for each mouse.

*Echocardiography*

Two-dimensional transthoracic echocardiography was performed on all animals under 1.5% inhaled isoflurane. Imaging was conducted using a 10–13 MHz phased array linear transducer (IL12i, GE Healthcare, Madrid, Spain) with a Vivid Q system (GE Healthcare, Madrid, Spain). The images were recorded and later analysed offline using EchoPac software (v.08.1.6, GE Healthcare, Madrid, Spain). Proximal aortic segments were examined in a parasternal long-axis view, and the AR diameter was measured from inner edge to inner edge at end-diastole at the level of the sinus of Valsalva. The average of three consecutive cardiac cycles was used for each measurement, with the operator being blinded to group assignment.

*Tissue histopathology and electron microscopy*

Paraffin-embedded tissue arrays (TMAs) of mice aortae and hearts from all experimental groups were transversally sectioned into 5-µm slices. Heart sections were stained with a regular haematoxylin-eosin protocol. Aorta sections were stained with Verhoeff–Van Gieson (Merck HT25A) following the supplier's recommendations. Images were obtained under visible light using a Leica Leitz DMRB microscope (10X or 20X) equipped with a Leica DC500 camera. All measurements were carried out blindly, with no knowledge of genotypes.

Elastic fibre ruptures were quantified by counting breaks >20 µm (large discontinuities) in the normal 360° circumferential continuity of each elastic lamina in the aortic media. These breaks were counted in four representative images from three non-consecutive sections of the same ascending aorta.

The undulation factor is defined as the ratio of elastic fibre size to the number of contact points where a continuous elastic fibre intersects or touches a virtually established straight line (value “=1” means that the fibre is completely straight; value “>1” indicates the undulation level of the fibre; the higher value, the greater the undulation).

For vascular smooth muscle cell (VSMC) density, aortic sections were deparaffinised and rehydrated before DAPI staining and mounted on the same glass slide using a fluorescence-compatible mounting medium. Images were obtained using a Widefield Leica AF6000 (40X oil immersion) equipped with a Leica DC500 camera. For electron microscopy, isolated ascending aortae from mice of the four experimental groups were cut into rings and fixed with 2.5% glutaraldehyde and 2% paraformaldehyde in 0.1 M phosphate buffer, postfixed with 1% OsO_4_, dehydrated with ethanol, properly oriented, and embedded in Spurr resin before sectioning with a Leica ultramicrotome UC7 (Leica Microsystems). Ultrathin sections were not counterstained and were directly observed under a JEOL JEM-1010 TEM fitted with a Gatan Orius SC1000 (model 832) digital camera.

*In-situ detection of superoxide anions*

Deparaffinised and rehydrated paraffin-embedded cardiac sections were stained with a fresh 5 µM dihydroethidium (DHE) staining solution (Invitrogen D1168). Slides were incubated in a dark humidified chamber at 37°C for 30-45 min to allow DHE penetration and interaction with superoxide anions. Fluorescence images were captured using a Widefield Leica AF6000 microscope equipped with the appropriate filters (excitation: 518 nm, emission: 605 nm for ethidium). Images were acquired from representative fields in each section.

*Western blotting*

Heart tissues were homogenised in RIPA buffer containing protease inhibitors (2 mM phenylmethylsulfonyl fluoride, 10 g/L aprotinin, 1 g/L leupeptin, and 1 g/L pepstatin) and phosphatase inhibitors (2 mM Na₃VO₄ and 100 mM NaF). Protein concentration was measured using a DC protein assay kit (Bio-Rad, Berkeley, CA, USA).

Nitrocellulose membranes (GE10600002 Amersham Protran, Amersham, UK) were incubated overnight at 4°C with the following primary antibodies: total OXPHOS rodent antibody cocktail (45-8099 Invitrogen; 1:5,000), TOM20 (SC-17764 Santa Cruz Biotechnology, Santa Cruz, CA, USA; 1:5,000), MMP-2 (ab37150 Abcam, Cambridge, UK; 1:1,000), and G-actin (A2066, Sigma; 1:10,000). Thereafter, membranes were rinsed and incubated with horse radish peroxidase (HRP)-conjugated secondary antibodies (W4011 (rabbit) or W021 (mouse) Promega, Madrid, Spain; 1:3,000), and protein bands were visualised using Western Blotting Luminol Reagent (Santa Cruz Biotechnology, Santa Cruz, CA, USA).

*Images Analysis*

All images were analysed using Fiji ImageJ Analysis software (ImageJ 1.54f, National Institute of Health, Bethesda, Maryland, USA).

*Statistical analysis*

Before conducting statistical analyses, data were tested for normality using the Shapiro-Wilk test. After the relevant test (parametric or not), the data were analysed to investigate potential differences due to sex. Where no significant sex-based differences were found, data were grouped based on genotype alone.

To compare genotypes, One-way ANOVA with Dunnet’s *post-hoc* test or Kruskal-Wallis test with Dunn’s post-test for multiple comparisons were used. To analyse progression over time, we used a Mixed-effects model (REML).

The statistical test applied in each case is indicated in each figure legend. Statistical details are shown in the Supplementary Tables S1-S7. Results are expressed as mean ± SEM. A *p*-value ≤0.05 was considered statistically significant. All statistical analyses and graph generation were performed using GraphPad Prism 9 software.

**SUPPLEMENTAL TABLES**

**Table S8. SUMMARY OF RESULTS IN MARFAN AND CD MOUSE MODELS AND PATIENTS**

|  | **Panel/Figure** | **MOUSE MODELS** | | | **PATIENTS** | |
| --- | --- | --- | --- | --- | --- | --- |
|  |  | **MFS** | **CD** | **CDMFS** | **MFS** | **WBS** |
| **Aortic root**  **(2 months)** | A | ↑↑ | ↓↓ | ↑↑ | Aneurysm and  Vascular stiffness | Stenosis and  Vascular stiffness |
| **Aortic root**  **(6 months)** | A | ↑↑ | ↓↓ | ↑↑ |  |  |
| **Elastic fibre breaks** | B | ↑↑↑↑ | Ø | ↑↑ | ↑↑↑ | Ø |
| **Undulation factor** | C | ↑↑↑↑ | Ø | ↑↑↑↑ | ? | ? |
| **Tunica**  **Media (%)** | B | ↑↑↑↑ | ↑↑↑↑ | ↑↑↑↑ | ↑↑ | ↑↑↑ |
| **VSMC**  **density** | SFig 2A,B | ↓ | ↑↑ | Ø | ↓↓ | ↑↑↑ |
| **Aortic**  **Area (%)** | SFig. 2A,B | ↑↑↑ | ↓↓↓↓ | ↓↓↓↓ | ↑ | Ø |
| **Blood pressure (SBP)** | D | Ø | ↑↑ | ↑↑↑ | Ø | ↑↑ |
| **Ejection fraction (EF)** | E | ↓↓↓ | ↓↓↓ | ↓↓↓ | Ø/↓ | ↓↓ |
| **Cardiac MMP2 protein levels** | G | ↑↑↑ | Ø | Ø | ? | ? |
| **Cardiac mitochondrial OXPHOS** | F | Ø | ↓↓↓↓ | Ø | ? | ? |
| **Cardiac mitochondrial morphology** | SFig.3 | Lightly altered | Very altered | Very altered | ? | ? |
| **Cardiac DHE/oxidative stress** | SFig. 4 | Ø | ↑↑↑ | Ø | ? | ? |

↑, augmented; ↓, reduced; Ø, no change; ?, unknown. The number of ↑↓is indicative of the observed outcome intensity. MFS, Marfan syndrome; WBS, Williams-Beuren syndrome; CD, complete deletion WBS mouse model; CDMFS, heterozygous mixed MFS and CD mouse model.

**SUPPLEMENTAL FIGURES**

**Supplementary Figure 1.** Body weight. Progression of weight over time in males (**A**) and females **(B).** All animals were weighed at two, three, four and six months of age. The weight of the animals was separately analysed by sex due to significant differences in this factor. Circles, WT; Squares, MFS; Triangles, CD; Diamonds, CDMFS. Data are expressed as the mean ± SEM. Statistical significance was calculated by two-way Mixed effect analysis followed by Dunnet’s comparison test. *Genotype effect; ^#^ time effect. ****^, ####^*p*<0.0001.

**Supplementary Figure 2.** Aortic histopathology. (**A**) Representative bright-field microscopy images of paraffin-embedded ascending aorta slides. Scale bar, 100 µm. (**B**) Histogram representing the area (tunica media plus the lumen) of the ascending aorta normalised to the mean area of WT littermates. (**C**) Histogram of vascular smooth muscle cells (VSMC) density (DAPI staining) relative to the mean density of WT littermates. Data are expressed as the mean ± SEM. Statistical significance was calculated using one-way ANOVA followed by Dunnett's multiple comparisons test. **p*<0.05; ***p*<0.01; ****p*<0.001; *****p*<0.0001.

**Supplementary Figure 3.** Cardiac mitochondrial ultrastructure. Ultrathin sections of WT, MFS, CD, and CDMFS heart ventricles showing the morphology and disposition of mitochondria between cardiac fibres. Insets show magnified images of some areas for a better visualisation of mitochondrial morphology.

**Supplementary Figure 4**. Cardiac redox stress. Dihydroethidium (DHE) was quantified by fluorescence in the left ventricles. Data are expressed as the mean ± SEM. Statistical significance was calculated using one-way ANOVA followed by Dunnett's multiple comparisons test. ****p*<0.001.

**SUPPLEMENTAL DISCUSSION**

In contrast to observed with the AR diameter, aortic elastic fibres in CDMFS mice presented the opposite result to MFS aorta and, therefore, a highly similar phenotype to CD, with an aortic wall with much more continuous elastic fibres almost devoid of elastic breaks, being closer to the WT aortic phenotype. Concomitantly to these observations, MFS and CDMFS elastic fibres were more undulated than in WT and CD, which correlates with non-pressurised aortae of the histological preparations (smaller diameter) compared with the dilatation presented in the ultrasound tests (intermediate pressure aortas under anaesthesia). These results also agree with previously reported mechanical studies in which elastin and fibrillin-1 were postulated to have distinct roles in vessel distensibility [4]. Ultrastructural analysis of the aortic media from the experimental groups shows that the CDMFS aorta has thinner elastic laminae compared with MFS aortae. In any case, the structural organisation of the media in MFS, CD, and CDMFS is invariably affected, in which VSMC clearly show altered morphology and a high degree of disconnection with adjacent fibres and collagen proliferation between cells and elastic fibres, which, in the case of MFS, explains the known elevated aortic stiffness [5,6]. All mutant models show thickening of the aortic tunica media compared with their WT counterparts. The cause of this thickening differs between models. On the one hand, in the MFS aorta, it is caused by the increased presence of proteoglycans and collagens that accompanies the reduction in VSMC [7,8]. Conversely, the main cause of increased media thickness in CD aortae is VSMC hyperplasia.

The characteristic elevated SBP observed in WBS patients [9] and CD mice [2] has always been attributed to supravalvular aortic stenosis (SVAS) due to elastin deficiency (*Eln^+/−^* mice are also hypertensive) [4,10]. We observed that double heterozygous CDMFS animals are also hypertensive. In line with previous data [4], our results support the idea that elastin plays a greater role than fibrillin-1 in establishing baseline blood pressure. In the same study, authors associated hypertension with a significant increase in left ventricular wet weight in double heterozygous *Eln^+/-^-Fbn1^+-/^; ELN/FBN)*, and with a remarkable trend towards the same increase in *Eln^+/−^* mice [4]. In our study, note that at a later age (six months) and despite MFS mice having normal SBP, *Fbn1* mutation contributed to the exacerbated SBP seen in CDMFS mice compared with CD animals. It is possible that the apparently “healthy” SBP of MFS mice (and perhaps in patients as well) could be considered “unhealthy” as the values obtained seem to predispose the animals to the altered cardiac parameters associated with hypertension. Note that EF was not particularly aggravated in CDMFS mice compared with CD and MFS, suggesting that there is no additive or synergistic damage between both elastin and fibrillin-1 haploinsufficiency in this respect.

Oxidative stress contributes to cardiovascular damage in rare genetic aortic diseases [11], and mitochondrial dysfunction is an important contributor to oxidative stress by increasing the production of ROS thought distinct and complexes mechanisms. To analyse mitochondrial functionality, we evaluated mitochondrial OXPHOS protein levels in mice hearts. In accordance with previous observations CD cardiomyocytes clearly showed OXPHOS reduction compared with their WT counterparts [13]. However, in contrast to reported in aortic aneurysm [12], mitochondrial dysfunction in MFS cardiomyocytes was not observed although a trend toward reduced OXPHOS was noted. Notably, CDMFS cardiomyocytes displayed an OXPHOS profile that closely resembled that of MFS rather than CD cardiomyocytes. Given the maternal inheritance of mitochondria, this similarity may reflect the mitochondrial origin from MFS mothers, whose mitochondrial function appears to be less affected than that observed in CD mice.

When redox stress was analysed with DHE marker, only CD mutant hearts showed significantly elevated values, while hearts of MFS and CDMFS animals presented values comparable to WT. Results from CDMFS suggest some protective cardiac anti-redox stress effect triggered by *FBN1* over *ELN* haploinsufficiency. This could be related to a higher endogenous antioxidant machinery response (e.g., NRF2) that is seriously damaged in CD cardiomyocytes [14].

Growing evidence indicates the existence of a bidirectional crosstalk between mitochondrial dysfunction, oxidative stress, and MMP2 regulation. It has been postulated that an indirect source of mitochondrial dysfunction could be MMP2 activation [15] [16], and that full-length MMP2 is activated during oxidative stress and mitochondrial dysfunction [17]. However, our results in mice hearts do not support this hypothesis, as we observed a trend of increase in MMP2 levels in the cardiac tissue in MFS and CDMFS, but not in CD, a pattern that do not correlate with that observed for mitochondrial OXPHOS and DHE marker. Although elevated MMP2 protein and altered mitochondrial activity levels have been described in the ascending aorta of the MFS model [12; 18], it cannot be discarded that their postulated association could be different in the heart and depending on the examined cardiomyopathy. Moreover, our observation that oxidative stress is elevated in CD but not in MFS and CDMFS hearts despite OXPHOS alterations in CD, suggest the existence of a genotype-specific differences in endogenous antioxidant mechanisms that may contribute to the divergent oxidative phenotypes observed. Clarifying this possibility, would require direct measurements of intrinsic antioxidant pathways in these mouse models, an issue that should be addressed in more specific and detailed future studies. Nonetheless, our data suggest that the cardiac dysfunction observed in CDMFS would be more attributable to fibrillin-1 than elastin haploinsufficiency.

**REFERENCES**

[1]	Kilkenny, C.; Browne, W.; Cuthill, I.; Emerson, M.; Altman, D.Improving Bioscience Research Reporting: The ARRIVE Guidelines for Reporting Animal Research. *J Pharmacol Pharmacother* **2010**, *1* (2), 94.

[2]	Segura-Puimedon, M.; Sahún, I.; Velot, E.; Dubus, P.; Borralleras, C.; Rodrigues, A. J.; Valero, M. C.; Valverde, O.; Sousa, N.; Herault, Y.; Dierssen, M.; Pérez-Jurado, L. A.; Campuzano, V. Heterozygous Deletion of the Williams–Beuren Syndrome Critical Interval in Mice Recapitulates Most Features of the Human Disorder. *Hum Mol Genet* **2014**, *23* (24), 6481–6494.

[3]	Borralleras, C.; Sahun, I.; Pérez-Jurado, L. A.; Campuzano, V.Intracisternal Gtf2i Gene Therapy Ameliorates Deficits in Cognition and Synaptic Plasticity of a Mouse Model of Williams–Beuren Syndrome. *Molecular Therapy* **2015**, *23* (11), 1691–1699.

[4]	Carta, L.; Wagenseil, J. E.; Knutsen, R. H.; Mariko, B.; Faury, G.; Davis, E. C.; Starcher, B.; Mecham, R. P.; Ramirez, F.Discrete Contributions of Elastic Fiber Components to Arterial Development and Mechanical Compliance. *Arterioscler Thromb Vasc Biol* **2009**, *29* (12), 2083–2089.

[5]	Cox, K.; Oquendo, Y. A.; Liang, D.; Selamet Tierney, E. S.Aortic Root Dilation in Adult Patients with Marfan Syndrome: Does Aortic Root Stiffness Matter? *JTCVS Open* **2022**, *10*, 113–120.

[6]	Bonfioli, G. B.; Rodella, L.; Rosati, R.; Carrozza, A.; Metra, M.; Vizzardi, E.Aortopathies: From Etiology to the Role of Arterial Stiffness. *J Clin Med* **2023**, *12* (12), 3949.

[7]	Hwang, C. T.; Halper, J.Proteoglycans and Diseases of Soft Tissues; **2021**; pp 127–138.

[8]	Ruiz-Rodríguez, M. J.; Oller, J.; Martínez-Martínez, S.; Alarcón-Ruiz, I.; Toral, M.; Sun, Y.; Colmenar, Á.; Méndez-Olivares, M. J.; López-Maderuelo, D.; Kern, C. B.; Nistal, J. F.; Evangelista, A.; Teixido-Tura, G.; Campanero, M. R.; Redondo, J. M.Versican Accumulation Drives Nos2 Induction and Aortic Disease in Marfan Syndrome via Akt Activation. *EMBO Mol Med* **2024**, *16* (1), 132–157.

[9]	Bouchireb, K.; Boyer, O.; Bonnet, D.; Brunelle, F.; Decramer, S.; Landthaler, G.; Liutkus, A.; Niaudet, P.; Salomon, R.Clinical Features and Management of Arterial Hypertension in Children with Williams-Beuren Syndrome. *Nephrology Dialysis Transplantation* **2010**, *25* (2), 434–438.

[10]	Brengle, B. M.; Lin, M.; Roth, R. A.; Jones, K. D.; Wagenseil, J. E.; Mecham, R. P.; Halabi, C. M.A New Mouse Model of Elastin Haploinsufficiency Highlights the Importance of Elastin to Vascular Development and Blood Pressure Regulation. *Matrix Biology* **2023**, *117*, 1–14.

[11]	Egea, G.; Jiménez-Altayó, F.; Campuzano, V.Reactive Oxygen Species and Oxidative Stress in the Pathogenesis and Progression of Genetic Diseases of the Connective Tissue. *Antioxidants* **2020**, *9* (10).

[12]	Oller, J.; Gabandé-Rodríguez, E.; Ruiz-Rodríguez, M. J.; Desdín-Micó, G.; Aranda, J. F.; Rodrigues-Diez, R.; Ballesteros-Martínez, C.; Blanco, E. M.; Roldan-Montero, R.; Acuña, P.; Forteza Gil, A.; Martín-López, C. E.; Nistal, J. F.; Lino Cardenas, C. L.; Lindsay, M. E.; Martín-Ventura, J. L.; Briones, A. M.; Miguel Redondo, J.; Mittelbrunn, M.Extracellular Tuning of Mitochondrial Respiration Leads to Aortic Aneurysm. *Circulation* **2021**, *143* (21), 2091–2109.

[13]	Abdalla, N.; Tobías-Baraja, E.; Gonzalez, A.; Garrabou, G.; Egea, G.; Campuzano, V.Dysfunctional Mitochondria in the Cardiac Fibers of a Williams–Beuren Syndrome Mouse Model. *Int J Mol Sci* **2023**, *24* (12), 10071.

[14]	Ortiz-Romero, P.; Borralleras, C.; Bosch-Morató, M.; Guivernau, B.; Albericio, G.; Muñoz, F. J.; Pérez-Jurado, L. A.; Campuzano, V. Epigallocatechin-3-Gallate Improves Cardiac Hypertrophy and Short-Term Memory Deficits in a Williams-Beuren Syndrome Mouse Model. *PLoS One* **2018**, *13* (3), e0194476.

[15]	Bassiouni, W.; Valencia, R.; Mahmud, Z.; Seubert, J. M.; Schulz, R.Matrix Metalloproteinase-2 Proteolyzes Mitofusin-2 and Impairs Mitochondrial Function during Myocardial Ischemia–Reperfusion Injury. *Basic Res Cardiol* **2023**, *118* (1), 29.

[16]	Hughes, B. G.; Schulz, R.Targeting MMP-2 to Treat Ischemic Heart Injury. *Basic Res Cardiol* **2014**, *109* (4), 424.

[17]	Viappiani, S.; Nicolescu, A. C.; Holt, A.; Sawicki, G.; Crawford, B. D.; León, H.; van Mulligen, T.; Schulz, R.Activation and Modulation of 72kDa Matrix Metalloproteinase-2 by Peroxynitrite and Glutathione. *Biochem Pharmacol* **2009**, *77* (5), 826–834.

[18]	Xiong, W.; Meisinger, T.; Knispel, R.; Worth, J. M.; Baxter, B. T.MMP-2 Regulates Erk1/2 Phosphorylation and Aortic Dilatation in Marfan Syndrome. *Circ Res* **2012**, *110* (12).
