# Supplementary material for: Differential contribution of elastin and fibrillin-1 to the cardiovascular phenotype of a double heterozygous Marfan and Williams-Beuren syndrome mouse model
Source: Genes Dis. 2026 Jun 1;14(2):102268. doi: 10.1016/j.gendis.2026.102268 (PMC13634561; doi:10.1016/j.gendis.2026.102268)
Supplement: Supplementary file 2 [file mmc2.docx]

**STATISTIC TABLES (Males** and **Females)**

**TABLE S1: VIABILITY AND BODY WEIGHT**

| **VIABILITY** | | | | | | | | | | | | | | | | | | | | | | | | | | | | | | | | | | | | | | | | | | | | |
| --- | --- | --- | --- | --- | --- | --- | --- | --- | --- | --- | --- | --- | --- | --- | --- | --- | --- | --- | --- | --- | --- | --- | --- | --- | --- | --- | --- | --- | --- | --- | --- | --- | --- | --- | --- | --- | --- | --- | --- | --- | --- | --- | --- | --- |
| **Genotype** | | | | | | **WT** | | | | | | | | | | **MFS** | | | | | | | | | **CD** | | | | | | | | | | | **CDMFS** | | | | | | | | |
| **Number of pups** | | | | | | 41 | | | | | | | | | | 36 | | | | | | | | | 32 | | | | | | | | | | | 21 | | | | | | | | |
| **Chi-square test** | | | | | | | | | | | | | | | | | | | | | | | | | | | | | | | | | | | | | | | | | | | | |
| **Chi-square** | | | | | | | | | | | | **DF** | | | | | | | | | | | | | | | | **P** | | | | | | | | | | | | | | | | |
| 7.016 | | | | | | | | | | | | 3 | | | | | | | | | | | | | | | | 0.0714 | | | | | | | | | | | | | | | | |
|  | | | | | | | | | | | | | | | | | | | | | | | | | | | | | | | | | | | | | | | | | | | | |
| **BODY WEIGHT (g) -MALES** | | | | | | | | | | | | | | | | | | | | | | | | | | | | | | | | | | | | | | | | | | | | |
| **Shapiro-Wilk test** | **WT-2M** | **WT-3M** | | **WT-4M** | | | | **WT-6M** | | **MFS-2M** | | | | | **MFS-3M** | | | **MFS-4M** | | **MFS-6M** | | | **CD-2M** | | | **CD-3M** | | | **CD-4M** | | | | **CD-6M** | | | | **CDMFS-2M** | | **CDMFS-3M** | | **CDMFS-4M** | | **CDMFS-6M** | |
| **W** | 0,9379 | 0,9046 | | 0,9212 | | | | 0,9788 | | 0,9753 | | | | | 0,9579 | | | 0,8787 | | 0,9152 | | | 0,9721 | | | 0,9190 | | | 0,9243 | | | | 0,9436 | | | | 0,8891 | | 0,8742 | | 0,9083 | | 0,9499 | |
| **P value** | 0,3916 | 0,2104 | | 0,3285 | | | | 0,9606 | | 0,9148 | | | | | 0,7455 | | | 0,1000 | | 0,2159 | | | 0,9074 | | | 0,3836 | | | 0,4295 | | | | 0,5935 | | | | 0,3789 | | 0,2837 | | 0,4572 | | 0,7398 | |
|  | | | | | | | | | | | | | | | | | | | | | | | | | | | | | | | | | | | | | | | | | | | | |
| **ONE- WAY ANOVA-2M** | | | | | | | | | | | | | | | | | | | | | | | | | | | | | | | | | | | | | | | | | | | | |
| **ANOVA table** | | | | | | | | | | | | | **F (DFn, DFd)** | | | | | | | | | | | | | | | | | | | **P value** | | | | | | | | | | | | |
| Genotype | | | | | | | | | | | | | F (3, 41) = 16,16 | | | | | | | | | | | | | | | | | | | P<0,0001 | | | | | | | | | | | | |
| **Dunnett's multiple comparisons test** | | | | | | | | | | | | | | | | | | | | | | **Adjusted P Value** | | | | | | | | | | | | | | | | | | | | | | |
| WT vs. MFS | | | | | | | | | | | | | | | | | | | | | | 0,8306 | | | | | | | | | | | | | | | | | | | | | | |
| WT vs. CD | | | | | | | | | | | | | | | | | | | | | | 0,0001 | | | | | | | | | | | | | | | | | | | | | | |
| WT vs. CDMFS | | | | | | | | | | | | | | | | | | | | | | 0,0002 | | | | | | | | | | | | | | | | | | | | | | |
| **ONE- WAY ANOVA-6M** | | | | | | | | | | | | | | | | | | | | | | | | | | | | | | | | | | | | | | | | | | | | |
| **ANOVA table** | | | | | | | | | | | | | **F (DFn, DFd)** | | | | | | | | | | | | | | | | | | | **P value** | | | | | | | | | | | | |
| Genotype | | | | | | | | | | | | | F (3, 40) = 11,39 | | | | | | | | | | | | | | | | | | | P<0,0001 | | | | | | | | | | | | |
| **Dunnett's multiple comparisons test** | | | | | | | | | | | | | | | | | | | | | | **Adjusted P Value** | | | | | | | | | | | | | | | | | | | | | | |
| WT vs. MFS | | | | | | | | | | | | | | | | | | | | | | 0,9997 | | | | | | | | | | | | | | | | | | | | | | |
| WT vs. CD | | | | | | | | | | | | | | | | | | | | | | 0,0001 | | | | | | | | | | | | | | | | | | | | | | |
| WT vs. CDMFS | | | | | | | | | | | | | | | | | | | | | | 0,0026 | | | | | | | | | | | | | | | | | | | | | | |
|  | | | | | | | | | | | | | | | | | | | | | | | | | | | | | | | | | | | | | | | | | | | | |
| **PROGRESSION** | | | | | | | | | | | | | | | | | | | | | | | | | | | | | | | | | | | | | | | | | | | | |
| **Mixed-effects model (REML)** | | | | | | | | | **F (DFn, DFd)** | | | | | | | | | | | | | | | | | | | | | | | | | **P value** | | | | | | | | | | |
| Time | | | | | | | | | F (1,847, 62,17) = 106,4 | | | | | | | | | | | | | | | | | | | | | | | | | <0,0001 | | | | | | | | | | |
| GENOTYPE | | | | | | | | | F (3, 44) = 53,28 | | | | | | | | | | | | | | | | | | | | | | | | | <0,0001 | | | | | | | | | | |
| Time x GENOTYPE | | | | | | | | | F (9, 101) = 0,8470 | | | | | | | | | | | | | | | | | | | | | | | | | 0,5750 | | | | | | | | | | |
|  | | | | | | | | | | | | | | | | | | | | | | | | | | | | | | | | | | | | | | | | | | | | |
| **BODY WEIGHT (g)-FEMALES** | | | | | | | | | | | | | | | | | | | | | | | | | | | | | | | | | | | | | | | | | | | | |
| **Shapiro-Wilk test** | | | **WT-2M** | | **WT-3M** | | **WT-4M** | | **WT-6M** | | | | | **MFS-2M** | | | **MFS-3M** | | **MFS-4M** | | **MFS-6M** | | | **CD-2M** | | | **CD-3M** | | | **CD-4M** | | | | | **CD-6M** | | | **CDMFS-2M** | | **CDMFS-3M** | | **CDMFS-4M** | | **CDMFS-6M** |
| W | | | 0,9661 | | 0,9066 | | 0,9077 | | 0,9671 | | | | | 0,9074 | | | 0,9387 | | 0,9402 | | 0,9617 | | | 0,9370 | | | 0,8769 | | | 0,9614 | | | | | 0,9227 | | | 0,9863 | | 0,9250 | | 0,9182 | | 0,9416 |
| **P value** | | | 0,8213 | | 0,2224 | | 0,0783 | | 0,7898 | | | | | 0,2636 | | | 0,6271 | | 0,6135 | | 0,8084 | | | 0,5512 | | | 0,0800 | | | 0,7891 | | | | | 0,4154 | | | 0,9843 | | 0,5089 | | 0,4557 | | 0,5984 |
|  | | | | | | | | | | | | | | | | | | | | | | | | | | | | | | | | | | | | | | | | | | | | |
| **ONE- WAY ANOVA-2M** | | | | | | | | | | | | | | | | | | | | | | | | | | | | | | | | | | | | | | | | | | | | |
| **ANOVA table** | | | | | | | | | | | **F (DFn, DFd)** | | | | | | | | | | | | | | | | | | | | **P value** | | | | | | | | | | | | | |
| Genotype | | | | | | | | | | | F (3, 36) = 12,51 | | | | | | | | | | | | | | | | | | | | P<0,0001 | | | | | | | | | | | | | |
| **Dunnett's multiple comparisons test** | | | | | | | | | | | | | | | | | | | | | | **Adjusted P Value** | | | | | | | | | | | | | | | | | | | | | | |
| WT vs. MFS | | | | | | | | | | | | | | | | | | | | | | 0,4724 | | | | | | | | | | | | | | | | | | | | | | |
| WT vs. CD | | | | | | | | | | | | | | | | | | | | | | 0,0022 | | | | | | | | | | | | | | | | | | | | | | |
| WT vs. CDMFS | | | | | | | | | | | | | | | | | | | | | | 0,0008 | | | | | | | | | | | | | | | | | | | | | | |
| **ONE- WAY ANOVA-6M** | | | | | | | | | | | | | | | | | | | | | | | | | | | | | | | | | | | | | | | | | | | | |
| **ANOVA table** | | | | | | | | | | | **F (DFn, DFd)** | | | | | | | | | | | | | | | | | | | | **P value** | | | | | | | | | | | | | |
| Genotype | | | | | | | | | | | F (3, 42) = 8,883 | | | | | | | | | | | | | | | | | | | | P=0,0001 | | | | | | | | | | | | | |
| **Dunnett's multiple comparisons test** | | | | | | | | | | | | | | | | | | | | | | **Adjusted P Value** | | | | | | | | | | | | | | | | | | | | | | |
| WT vs. MFS | | | | | | | | | | | | | | | | | | | | | | 0,3771 | | | | | | | | | | | | | | | | | | | | | | |
| WT vs. CD | | | | | | | | | | | | | | | | | | | | | | <0,0001 | | | | | | | | | | | | | | | | | | | | | | |
| WT vs. CDMFS | | | | | | | | | | | | | | | | | | | | | | 0,0044 | | | | | | | | | | | | | | | | | | | | | | |
|  | | | | | | | | | | | | | | | | | | | | | | | | | | | | | | | | | | | | | | | | | | | | |
| **PROGRESSION** | | | | | | | | | | | | | | | | | | | | | | | | | | | | | | | | | | | | | | | | | | | | |
| **Mixed-effects model (REML)** | | | | | | | | | | | **F (DFn, DFd)** | | | | | | | | | | | | | | | | | | | | **P value** | | | | | | | | | | | | | |
| Time | | | | | | | | | | | F (2,142, 74,27) = 98,19 | | | | | | | | | | | | | | | | | | | | <0,0001 | | | | | | | | | | | | | |
| GENOTYPE | | | | | | | | | | | F (3, 47) = 24,67 | | | | | | | | | | | | | | | | | | | | <0,0001 | | | | | | | | | | | | | |
| Time x GENOTYPE | | | | | | | | | | | F (9, 104) = 1,094 | | | | | | | | | | | | | | | | | | | | 0,3741 | | | | | | | | | | | | | |
|  | | | | | | | | | | | | | | | | | | | | | | | | | | | | | | | | | | | | | | | | | | | | |

**TABLE S2: AORTIC ROOT DIAMETER**

| **AORTIC ROOT** | | | | | | | | | | | | | | | | | | | | | | | | | | | |
| --- | --- | --- | --- | --- | --- | --- | --- | --- | --- | --- | --- | --- | --- | --- | --- | --- | --- | --- | --- | --- | --- | --- | --- | --- | --- | --- | --- |
| **Shapiro-Wilk test** | **WT-2M** | **MFS-2M** | **CD-2M** | | | **CDMFS-2M** | **WT-F-2** | | **MFS-F-2** | | | **CD-F-2** | **CDMFS-F-2** | | **WT-6M** | **MFS-6M** | | | **CD-6M** | | **CDMFS-6M** | **WT-F-6** | | | **MFS-F-6** | **CD-F-6** | **CDMFS-F-6** |
| W | 0,9430 | 0,9131 | 0,9145 | | | 0,9410 | 0,9460 | | 0,9670 | | | 0,9104 | 0,9190 | | 0,9593 | 0,9603 | | | 0,9173 | | 0,9599 | 0,9426 | | | 0,9040 | 0,9144 | 0,8122 |
| **P value** | 0,2982 | 0,0846 | 0,1829 | | | 0,5928 | 0,6218 | | 0,8683 | | | 0,4389 | 0,5314 | | 0,3787 | 0,4696 | | | 0,2303 | | 0,7970 | 0,4528 | | | 0,1291 | 0,4946 | 0,1259 |
| **THREE-WAY ANOVA SEX-GENOTYPE-TIME EFFECT** | | | | | | | | | | | | | | | | | | | | | | | | | | | |
| **Three-way ANOVA** | | | | | | | | **F (DFn, DFd)** | | | | | | | | | | | | **P value** | | | | | | | |
| GENOTYPE | | | | | | | | F (3, 112) = 92,48 | | | | | | | | | | | | P<0,0001 | | | | | | | |
| TIME | | | | | | | | F (1, 112) = 93,77 | | | | | | | | | | | | P<0,0001 | | | | | | | |
| SEX | | | | | | | | F (1, 112) = 0,9959 | | | | | | | | | | | | P=0,3205 | | | | | | | |
| GENOTYPE x TIME | | | | | | | | F (3, 112) = 1,298 | | | | | | | | | | | | P=0,2787 | | | | | | | |
| GENOTYPE x SEX | | | | | | | | F (3, 112) = 1,268 | | | | | | | | | | | | P=0,2890 | | | | | | | |
| TIME x SEX | | | | | | | | F (1, 112) = 0,02473 | | | | | | | | | | | | P=0,8753 | | | | | | | |
| GENOTYPE x TIME x SEX | | | | | | | | F (3, 112) = 0,1862 | | | | | | | | | | | | P=0,9056 | | | | | | | |
|  | | | | | | | | | | | | | | | | | | | | | | | | | | | |
| **Shapiro-Wilk test** | | | | **WT-2M** | | | | | | **MFS-2M** | | | | | | | **CD-2M** | | | | | | | **CDMFS-2M** | | | |
| W | | | | 0,9430 | | | | | | 0,9131 | | | | | | | 0,9145 | | | | | | | 0,9410 | | | |
| **P value** | | | | 0,2982 | | | | | | 0,0846 | | | | | | | 0,1829 | | | | | | | 0,5928 | | | |
| **ONE- WAY ANOVA-2M** | | | | | | | | | | | | | | | | | | | | | | | | | | | |
| **ANOVA table** | | | | | | | | **F (DFn, DFd)** | | | | | | | | | | | | **P value** | | | | | | | |
| Genotypes | | | | | | | | F (3, 57) = 57,86 | | | | | | | | | | | | P<0,0001 | | | | | | | |
| **Dunnett's multiple comparisons test** | | | | | | | | | | | | | | **Adjusted P Value** | | | | | | | | | | | | | |
| WT vs. MFS | | | | | | | | | | | | | | <0,0001 | | | | | | | | | | | | | |
| WT vs. CD | | | | | | | | | | | | | | <0,0001 | | | | | | | | | | | | | |
| WT vs. CDMFS | | | | | | | | | | | | | | 0,0001 | | | | | | | | | | | | | |
|  | | | | | | | | | | | | | | | | | | | | | | | | | | | |
| **Shapiro-Wilk test** | | | | | **WT-6M** | | | | | | **MFS-6M** | | | | | | | **CD-6M** | | | | | **CDMFS-6M** | | | | |
| W | | | | | 0,9593 | | | | | | 0,9603 | | | | | | | 0,9173 | | | | | 0,8678 | | | | |
| **P value** | | | | | 0,3787 | | | | | | 0,4696 | | | | | | | 0,2303 | | | | | 0,0943 | | | | |
| **ONE- WAY ANOVA-6M** | | | | | | | | | | | | | | | | | | | | | | | | | | | |
| **ANOVA table** | | | | | | | | **F (DFn, DFd)** | | | | | | | | | | | | **P value** | | | | | | | |
| Genotypes | | | | | | | | F (3, 68) = 34,23 | | | | | | | | | | | | P<0,0001 | | | | | | | |
| **Dunnett's multiple comparisons test** | | | | | | | | | | | | | | **Adjusted P Value** | | | | | | | | | | | | | |
| WT vs. MFS | | | | | | | | | | | | | | <0,0001 | | | | | | | | | | | | | |
| WT vs. CD | | | | | | | | | | | | | | <0,0001 | | | | | | | | | | | | | |
| WT vs. CDMFS | | | | | | | | | | | | | | <0,0001 | | | | | | | | | | | | | |
|  | | | | | | | | | | | | | | | | | | | | | | | | | | | |
| **PROGRESSION** | | | | | | | | | | | | | | | | | | | | | | | | | | | |
| **Mixed-effects model (REML)** | | | | | | | | **F (DFn, DFd)** | | | | | | | | | | | | **P value** | | | | | | | |
| Time | | | | | | | | F (1, 125) = 80,24 | | | | | | | | | | | | <0,0001 | | | | | | | |
| Genotype | | | | | | | | F (3, 125) = 74,88 | | | | | | | | | | | | <0,0001 | | | | | | | |
| Time x Genotype | | | | | | | | F (3, 125) = 0,6786 | | | | | | | | | | | | 0,5667 | | | | | | | |

**TABLE S3: ELASTIC LAMINA INTEGRITY AND VASCULAR HISTOLOGY**

| **TOTAL AREA** | | | | | | | | | | | | | | | | | | | | | | | | | | | | | | | | | | | | | | | | | | | | |
| --- | --- | --- | --- | --- | --- | --- | --- | --- | --- | --- | --- | --- | --- | --- | --- | --- | --- | --- | --- | --- | --- | --- | --- | --- | --- | --- | --- | --- | --- | --- | --- | --- | --- | --- | --- | --- | --- | --- | --- | --- | --- | --- | --- | --- |
| **Shapiro-Wilk test** | | | **WT-M** | | | | | | **MFS-M** | **CD-M** | | | | | | | | | | | | **CDMFS-M** | | | | | | **WT-F** | | | | | | | | **MFS-F** | | | | | | **CD-F** | | **CDMFS-F** |
| W | | | 0,9781 | | | | | | 0,8625 | 0,9298 | | | | | | | | | | | | 0,8720 | | | | | | 0,9392 | | | | | | | | 0,9455 | | | | | | 0,9158 | | 0,9163 |
| **P value** | | | 0,9374 | | | | | | 0,0620 | 0,4458 | | | | | | | | | | | | 0,1292 | | | | | | 0,6036 | | | | | | | | 0,7036 | | | | | | 0,5033 | | 0,5066 |
| **TWO-WAY ANOVA SEX-GENOTYPE** | | | | | | | | | | | | | | | | | | | | | | | | | | | | | | | | | | | | | | | | | | | | |
| **ANOVA table** | | | | | | | | | | | | | | | | **F (DFn, DFd)** | | | | | | | | | | | | | | | | | | | **P value** | | | | | | | | | |
| **Interaction** | | | | | | | | | | | | | | | | F (3, 39) = 0,9077 | | | | | | | | | | | | | | | | | | | P=0,4461 | | | | | | | | | |
| **Sex** | | | | | | | | | | | | | | | | F (1, 39) = 2,352 | | | | | | | | | | | | | | | | | | | P=0,1332 | | | | | | | | | |
| **Genotype** | | | | | | | | | | | | | | | | F (3, 39) = 37,00 | | | | | | | | | | | | | | | | | | | P<0,0001 | | | | | | | | | |
|  | | | | | | | | | | | | | | | | | | | | | | | | | | | | | | | | | | | | | | | | | | | | |
| **Shapiro-Wilk test** | | | | | | **WT** | | | | | | | | | | | | **MFS** | | | | | | | | | | | | | **CD** | | | | | | | | | | **CDMFS** | | | |
| W | | | | | | 0,9781 | | | | | | | | | | | | 0,8625 | | | | | | | | | | | | | 0,9298 | | | | | | | | | | 0,8720 | | | |
| **P value** | | | | | | 0,9374 | | | | | | | | | | | | 0,0620 | | | | | | | | | | | | | 0,4458 | | | | | | | | | | 0,1292 | | | |
| **ORDINARY ONE WAY ANOVA** | | | | | | | | | | | | | | | | | | | | | | | | | | | | | | | | | | | | | | | | | | | | |
| **ANOVA table** | | | | | | | | | | | **F (DFn, DFd)** | | | | | | | | | | | | | | | | | | | | | | | | **P value** | | | | | | | | | |
| Genotypes | | | | | | | | | | | F (3, 43) = 36,78 | | | | | | | | | | | | | | | | | | | | | | | | P<0,0001 | | | | | | | | | |
| **Dunnett's multiple comparisons test** | | | | | | | | | | | | | | | | | | | | | | | | | **Adjusted P Value** | | | | | | | | | | | | | | | | | | | |
| WT vs. MFS | | | | | | | | | | | | | | | | | | | | | | | | | 0,0003 | | | | | | | | | | | | | | | | | | | |
| WT vs. CD | | | | | | | | | | | | | | | | | | | | | | | | | <0,0001 | | | | | | | | | | | | | | | | | | | |
| WT vs. CDMFS | | | | | | | | | | | | | | | | | | | | | | | | | <0,0001 | | | | | | | | | | | | | | | | | | | |
|  | | | | | | | | | | | | | | | | | | | | | | | | | | | | | | | | | | | | | | | | | | | | |
| **TUNICA MEDIA (%)** | | | | | | | | | | | | | | | | | | | | | | | | | | | | | | | | | | | | | | | | | | | | |
| **Shapiro-Wilk test** | **WT-M** | | | | | | | **MFS-M** | | | | | | | **CD-M** | | | | | **CDMFS-M** | | | | | | | **WT-F** | | | | | | | | **MFS-F** | | | **CD-F** | | | | | | **CDMFS-F** |
| W | 0,9186 | | | | | | | 0,9142 | | | | | | | 0,9301 | | | | | 0,9360 | | | | | | | 0,8637 | | | | | | | | 0,9214 | | | 0,8079 | | | | | | 0,8844 |
| **P value** | 0,1397 | | | | | | | 0,2731 | | | | | | | 0,4824 | | | | | 0,5407 | | | | | | | 0,1308 | | | | | | | | 0,5156 | | | 0,0939 | | | | | | 0,3297 |
| **TWO-WAY ANOVA SEX-GENOTYPE** | | | | | | | | | | | | | | | | | | | | | | | | | | | | | | | | | | | | | | | | | | | | |
| **ANOVA table** | | | | | | | | | | | | | | **F (DFn, DFd)** | | | | | | | | | | | | | | | | | | | | **P value** | | | | | | | | | | |
| **Interaction** | | | | | | | | | | | | | | F (3, 39) = 0,5437 | | | | | | | | | | | | | | | | | | | | P=0,6553 | | | | | | | | | | |
| **Sex** | | | | | | | | | | | | | | F (1, 39) = 1,773 | | | | | | | | | | | | | | | | | | | | P=0,1908 | | | | | | | | | | |
| **Genotype** | | | | | | | | | | | | | | F (3, 39) = 19,08 | | | | | | | | | | | | | | | | | | | | P<0,0001 | | | | | | | | | | |
|  | | | | | | | | | | | | | | | | | | | | | | | | | | | | | | | | | | | | | | | | | | | | |
| **Shapiro-Wilk test** | | | | | **WT** | | | | | | | | | | | | **MFS** | | | | | | | | | | | | | **CD** | | | | | | | | | **CDMFS** | | | | | |
| W | | | | | 0,9186 | | | | | | | | | | | | 0,9142 | | | | | | | | | | | | | 0,9301 | | | | | | | | | 0,9360 | | | | | |
| **P value** | | | | | 0,1397 | | | | | | | | | | | | 0,2731 | | | | | | | | | | | | | 0,4824 | | | | | | | | | 0,5407 | | | | | |
| **ORDINARY ONE WAY ANOVA** | | | | | | | | | | | | | | | | | | | | | | | | | | | | | | | | | | | | | | | | | | | | |
| **ANOVA table** | | | | | | | | | | | | **F (DFn, DFd)** | | | | | | | | | | | | | | | | | | | | **P value** | | | | | | | | | | | | |
| Genotypes | | | | | | | | | | | | F (3, 42) = 24,33 | | | | | | | | | | | | | | | | | | | | P<0,0001 | | | | | | | | | | | | |
| **Dunnett's multiple comparisons test** | | | | | | | | | | | | | | | | | | | | | | | **Adjusted P Value** | | | | | | | | | | | | | | | | | | | | | |
| WT vs. MFS | | | | | | | | | | | | | | | | | | | | | | | <0,0001 | | | | | | | | | | | | | | | | | | | | | |
| WT vs. CD | | | | | | | | | | | | | | | | | | | | | | | <0,0001 | | | | | | | | | | | | | | | | | | | | | |
| WT vs. CDMFS | | | | | | | | | | | | | | | | | | | | | | | <0,0001 | | | | | | | | | | | | | | | | | | | | | |
|  | | | | | | | | | | | | | | | | | | | | | | | | | | | | | | | | | | | | | | | | | | | | |
| **VSMC DENSITY** | | | | | | | | | | | | | | | | | | | | | | | | | | | | | | | | | | | | | | | | | | | | |
| **Shapiro-Wilk test** | | **WT-M** | | | | | **MFS-M** | | | | | | **CD-M** | | | | | | | | **CDMFS-M** | | | | | **WT-F** | | | | | | | **MFS-F** | | | | **CD-F** | | | | | | **CDMFS-F** | |
| W | | 0,9469 | | | | | 0,9391 | | | | | | 0,9596 | | | | | | | | 0,9551 | | | | | 0,9375 | | | | | | | 0,9240 | | | | 0,8899 | | | | | | 0,8959 | |
| **P value** | | 0,2971 | | | | | 0,3074 | | | | | | 0,6860 | | | | | | | | 0,6777 | | | | | 0,5557 | | | | | | | 0,4630 | | | | 0,2336 | | | | | | 0,2651 | |
| **TWO-WAY ANOVA SEX-GENOTYPE** | | | | | | | | | | | | | | | | | | | | | | | | | | | | | | | | | | | | | | | | | | | | |
| **ANOVA table** | | | | | | | | | | | | | **F (DFn, DFd)** | | | | | | | | | | | | | | | | | | | | **P value** | | | | | | | | | | | |
| Interaction | | | | | | | | | | | | | F (3, 59) = 1,195 | | | | | | | | | | | | | | | | | | | | P=0,3194 | | | | | | | | | | | |
| SEX | | | | | | | | | | | | | F (1, 59) = 0,3792 | | | | | | | | | | | | | | | | | | | | P=0,5404 | | | | | | | | | | | |
| GENOTYPE | | | | | | | | | | | | | F (3, 59) = 10,53 | | | | | | | | | | | | | | | | | | | | P<0,0001 | | | | | | | | | | | |
|  | | | | | | | | | | | | | | | | | | | | | | | | | | | | | | | | | | | | | | | | | | | | |
| **Shapiro-Wilk test** | | | | **WT** | | | | | | | | | | | | | | | **MFS** | | | | | | | | | | **CD** | | | | | | | | | | | **CDMFS** | | | | |
| W | | | | 0,9469 | | | | | | | | | | | | | | | 0,9391 | | | | | | | | | | 0,9596 | | | | | | | | | | | 0,9551 | | | | |
| **P value** | | | | 0,2971 | | | | | | | | | | | | | | | 0,3074 | | | | | | | | | | 0,6860 | | | | | | | | | | | 0,6777 | | | | |
| **ORDINARY ONE WAY ANOVA** | | | | | | | | | | | | | | | | | | | | | | | | | | | | | | | | | | | | | | | | | | | | |
| **ANOVA table** | | | | | | | | | | | | | **F (DFn, DFd)** | | | | | | | | | | | | | | | | | | | | **P value** | | | | | | | | | | | |
| Genotypes | | | | | | | | | | | | | F (3, 62) = 9,955 | | | | | | | | | | | | | | | | | | | | P<0,0001 | | | | | | | | | | | |
| **Dunnett's multiple comparisons test** | | | | | | | | | | | | | | | | | | | | | | | | **Adjusted P Value** | | | | | | | | | | | | | | | | | | | | |
| WT vs. MFS | | | | | | | | | | | | | | | | | | | | | | | | 0,0372 | | | | | | | | | | | | | | | | | | | | |
| WT vs. CD | | | | | | | | | | | | | | | | | | | | | | | | 0,0078 | | | | | | | | | | | | | | | | | | | | |
| WT vs. CDMFS | | | | | | | | | | | | | | | | | | | | | | | | 0,6550 | | | | | | | | | | | | | | | | | | | | |
|  | | | | | | | | | | | | | | | | | | | | | | | |  | | | | | | | | | | | | | | | | | | | | |

| **NUMBER OF BREAKS** | | | | | | | | | | | | | |
| --- | --- | --- | --- | --- | --- | --- | --- | --- | --- | --- | --- | --- | --- |
| **Shapiro-Wilk test** | **WT-M** | | **MFS-M** | **CD-M** | | **CDMFS-M** | | **WT-F** | | **MFS-F** | **CD-F** | | **CDMFS-F** |
| W | 0,7875 | | 0,9132 | 0,8944 | | 0,8527 | | 0,8455 | | 0,9112 | 0,9714 | | 0,8950 |
| **P value** | 0,0006 | | 0,2026 | 0,1577 | | 0,0309 | | 0,0513 | | 0,4747 | 0,8500 | | 0,2601 |
| **Kruskal-Wallis test** | | | | | | | | | | | | | |
| Kruskal-Wallis statistic | | | | | | | 46.11 | | | | | | |
| Number of groups | | | | | | | 8 | | | | | | |
| P value | | | | | | | P<0,0001 | | | | | | |
| Dunn's multiple comparisons test | | | | | | | Adjusted P Value | | | | | | |
| WT-Male vs. WT-Female | | | | | | | >0,9999 | | | | | | |
| MFS-Male vs. MFS-Female | | | | | | | >0,9999 | | | | | | |
| CD-Male vs. CD-Female | | | | | | | >0,9999 | | | | | | |
| CDMFS-Male vs. CDMFS-Female | | | | | | | >0,9999 | | | | | | |
|  | | | | | | | | | | | | | |
| **Shapiro-Wilk test** | | **WT** | | | **MFS** | | | | **CD** | | | **CDMFS** | |
| W | | 0,7875 | | | 0,9132 | | | | 0,8944 | | | 0,8527 | |
| **P value** | | 0,0006 | | | 0,2026 | | | | 0,1577 | | | 0,0309 | |
| **Kruskal-Wallis test** | | | | | | | | | | | | | |
| **Kruskal-Wallis statistic** | | | | | | | 34.09 | | | | | | |
| **Number of groups** | | | | | | | 4 | | | | | | |
| **P value** | | | | | | | P<0,0001 | | | | | | |
| **Dunn's multiple comparisons test** | | | | | | | **Adjusted P Value** | | | | | | |
| WT vs. MFS | | | | | | | <0,0001 | | | | | | |
| WT vs. CD | | | | | | | 0,3907 | | | | | | |
| WT vs. CDMFS | | | | | | | 0,0014 | | | | | | |
|  | | | | | | | | | | | | | |
| **UNDULATION FACTOR** | | | | | | | | | | | | | |
| **Shapiro-Wilk test** | **WT-M** | | **MFS-M** | **CD-M** | | **CDMFS-M** | | **WT-F** | | **MFS-F** | **CD-F** | | **CDMFS-F** |
| W | 0,8947 | | 0,7861 | 0,9044 | | 0,9537 | | 0,8888 | | 0,9293 | 0,8528 | | 0,9037 |
| **P value** | 0,1355 | | 0,0203 | 0,3583 | | 0,7638 | | 0,1939 | | 0,5451 | 0,2481 | | 0,3541 |
| **Kruskal-Wallis test** | | | | | | | | | | | | | |
| Kruskal-Wallis statistic | | | | | | | 35.02 | | | | | | |
| Number of groups | | | | | | | 8 | | | | | | |
| P value | | | | | | | P<0,0001 | | | | | | |
| Dunn's multiple comparisons test | | | | | | | Adjusted P Value | | | | | | |
| WT-Male vs. WT-Female | | | | | | | >0,9999 | | | | | | |
| MFS-Male vs. MFS-Female | | | | | | | >0,9999 | | | | | | |
| CD-Male vs. CD-Female | | | | | | | 0,5606 | | | | | | |
| CDMFS-Male vs. CDMFS-Female | | | | | | | >0,9999 | | | | | | |
|  | | | | | | | | | | | | | |
| **Shapiro-Wilk test** | | **WT** | | | **MFS** | | | | **CD** | | | **CDMFS** | |
| W | | 0,9779 | | | 0,8913 | | | | 0,9287 | | | 0,9578 | |
| **P value** | | 0,8925 | | | 0,0702 | | | | 0,4355 | | | 0,7523 | |
| **ORDINARY ONE WAY ANOVA** | | | | | | | | | | | | | |
| **ANOVA table** | | | | **F (DFn, DFd)** | | | | | | **P value** | | | |
| Genotypes | | | | F (3, 54) = 23,67 | | | | | | P<0,0001 | | | |
| **Dunnett's multiple comparisons test** | | | | | | | **Adjusted P Value** | | | | | | |
| WT vs. MFS | | | | | | | <0,0001 | | | | | | |
| WT vs. CD | | | | | | | 0,9966 | | | | | | |
| WT vs. CDMFS | | | | | | | <0,0001 | | | | | | |
|  | | | | | | | | | | | | | |

**TABLE S4:SYSTOLIC BLOOD PRESSURE**

| **SYSTOLIC BLOOD PRESSURE** | | | | | | | | | | | | | | | | | | | | | | | | | | | |
| --- | --- | --- | --- | --- | --- | --- | --- | --- | --- | --- | --- | --- | --- | --- | --- | --- | --- | --- | --- | --- | --- | --- | --- | --- | --- | --- | --- |
| **Shapiro-Wilk test** | | **WT-M-2M** | | **WT-M-6M** | **MFS-M-2M** | **MFS-M-6M** | | **CD-M-2M** | **CD-M-6M** | | **CDMFS-M-2M** | | **CDMFS-M-6M** | | **WT-F-2M** | | **WT-F-6M** | **MFS-F-2M** | | | | **MFS-F-6M** | **CD-F-2M** | | **CD-F-6M** | **CDMFS-F-2M** | **CDMFS-F-6M** |
| W | | 0,954 | | 0,882 | 0,940 | 0,943 | | 0,901 | 0,884 | | 0,830 | | 0,954 | | 0,956 | | 0,951 | 0,878 | | | | 0,907 | 0,962 | | 0,931 | 0,840 | 0,887 |
| **P value** | | 0,719 | | 0,239 | 0,460 | 0,567 | | 0,191 | 0,208 | | 0,139 | | 0,768 | | 0,726 | | 0,614 | 0,098 | | | | 0,301 | 0,839 | | 0,594 | 0,130 | 0,345 |
|  |  | | | | | | | | | | | | | | | | | | | | | | | | | | |
| **THREE-WAY-ANOVA: SEX/GENOTYPE/TIME** | | | | | | | | | | | | | | | | | | | | | | | | | | | |
| **Mixed-effects model (REML)** | | | | | | | | **F (DFn, DFd)** | | | | | | | | | | | **P value** | | | | | | | | |
| GENOTYPE | | | | | | | | F (3, 125) = 67,05 | | | | | | | | | | | <0,0001 | | | | | | | | |
| TIME | | | | | | | | F (1, 125) = 53,64 | | | | | | | | | | | <0,0001 | | | | | | | | |
| SEX | | | | | | | | F (1, 125) = 0,2007 | | | | | | | | | | | 0,6549 | | | | | | | | |
| GENOTYPE x TIME | | | | | | | | F (3, 125) = 0,2608 | | | | | | | | | | | 0,8535 | | | | | | | | |
| GENOTYPE x SEX | | | | | | | | F (3, 125) = 0,6239 | | | | | | | | | | | 0,6009 | | | | | | | | |
| TIME x SEX | | | | | | | | F (1, 125) = 0,2790 | | | | | | | | | | | 0,5983 | | | | | | | | |
| GENOTYPE x TIME x SEX | | | | | | | | F (3, 125) = 1,255 | | | | | | | | | | | 0,2928 | | | | | | | | |
|  | | | | | | | | | | | | | | | | | | | | | | | | | | | |
| **Shapiro-Wilk test** | | | **WT-2M** | | | | **WT-6M** | | | **MFS-2M** | | **MFS-6M** | | | | **CD-2M** | | | | **CD-6M** | | | | **CDMFS-2M** | | **CDMFS-6M** | |
| W | | | 0,9747 | | | | 0,9655 | | | 0,9405 | | 0,9672 | | | | 0,9291 | | | | 0,8995 | | | | 0,9093 | | 0,9340 | |
| **P value** | | | 0,8162 | | | | 0,6585 | | | 0,1674 | | 0,6950 | | | | 0,1871 | | | | 0,1111 | | | | 0,2394 | | 0,4239 | |
|  | **SBP-2M** | | | | | | | | | | | | | | | | | | | | | | | | | | |
| **ANOVA table** | | | | | | | | **F (DFn, DFd)** | | | | | | | | | | | **P value** | | | | | | | | |
| Genotypes | | | | | | | | F (3, 54) = 8,941 | | | | | | | | | | | P<0,0001 | | | | | | | | |
| **Dunnett's multiple comparisons test** | | | | | | | | | | | | | | **Adjusted P Value** | | | | | | | | | | | | | |
| WT vs. MFS | | | | | | | | | | | | | | 0,9894 | | | | | | | | | | | | | |
| WT vs. CD | | | | | | | | | | | | | | 0,0050 | | | | | | | | | | | | | |
| WT vs. CDMFS | | | | | | | | | | | | | | 0,0014 | | | | | | | | | | | | | |
|  | | | | | | | | | | | | | | | | | | | | | | | | | | | |
| **SBP-6M** | | | | | | | | | | | | | | | | | | | | | | | | | | | |
| **ANOVA table** | | | | | | | | **F (DFn, DFd)** | | | | | | | | | | | **P value** | | | | | | | | |
| Genotypes | | | | | | | | F (3, 62) = 35,93 | | | | | | | | | | | P<0,0001 | | | | | | | | |
| **Dunnett's multiple comparisons test** | | | | | | | | | | | | | **Adjusted P Value** | | | | | | | | | | | | | | |
| WT vs. MFS | | | | | | | | | | | | | 0,2667 | | | | | | | | | | | | | | |
| WT vs. CD | | | | | | | | | | | | | <0,0001 | | | | | | | | | | | | | | |
| WT vs. CDMFS | | | | | | | | | | | | | <0,0001 | | | | | | | | | | | | | | |
|  | | | | | | | | | | | | | | | | | | | | | | | | | | | |
| **PROGRESSION** | | | | | | | | | | | | | | | | | | | | | | | | | | | |
| **Mixed-effects model (REML)** | | | | | | | | **F (DFn, DFd)** | | | | | | | | | | | | | **P value** | | | | | | |
| Time | | | | | | | | F (1, 61) = 67,24 | | | | | | | | | | | | | <0,0001 | | | | | | |
| Genotype | | | | | | | | F (3, 72) = 59,87 | | | | | | | | | | | | | <0,0001 | | | | | | |
| Time x Genotype | | | | | | | | F (3, 61) = 0,2325 | | | | | | | | | | | | | 0,8734 | | | | | | |

**TABLE S5: EJECTION FRACTION**

| **% EJECTION FRACTION (EF)** | | | | | | | | | | | | | | | | | | | | | | | | | | | | | | | |
| --- | --- | --- | --- | --- | --- | --- | --- | --- | --- | --- | --- | --- | --- | --- | --- | --- | --- | --- | --- | --- | --- | --- | --- | --- | --- | --- | --- | --- | --- | --- | --- |
| **Shapiro-Wilk test** | **WT-2M** | | **MFS-2M** | **CD-2M** | | **CDMFS-2M** | **WT-6M** | | | | | **MFS-6M** | **CD-6M** | | **CDMFS-6M** | | **WT-2M** | | **MFS-2M** | **CD-2M** | | | | | **CDMFS-2M** | **WT-6M** | | **MFS-6M** | **CD-6M** | | **CDMFS-6M** |
| W | 0,9809 | | 0,8841 | 0,9557 | | 0,9915 | 0,9339 | | | | | 0,9740 | 0,9415 | | 0,9089 | | 0,9698 | | 0,9762 | 0,8882 | | | | | 0,7991 | 0,8811 | | 0,9076 | 0,8243 | | 0,9294 |
| **P value** | 0,9709 | | 0,2454 | 0,7174 | | 0,9847 | 0,6231 | | | | | 0,9179 | 0,5975 | | 0,4768 | | 0,8889 | | 0,9417 | 0,2656 | | | | | 0,0797 | 0,1073 | | 0,2994 | 0,1260 | | 0,5456 |
| **THREE-WAY ANOVA SEX-GENOTYPE-TIME EFFECT** | | | | | | | | | | | | | | | | | | | | | | | | | | | | | | | |
| **Mixed-effects model (REML)** | | | | | | | | | | | **F (DFn, DFd)** | | | | | | | | | | | | | **P value** | | | | | | | |
| GENOTYPE | | | | | | | | | | | F (3, 105) = 16,61 | | | | | | | | | | | | | <0,0001 | | | | | | | |
| TIME | | | | | | | | | | | F (1, 105) = 13,85 | | | | | | | | | | | | | 0,0003 | | | | | | | |
| SEX | | | | | | | | | | | F (1, 105) = 1,342 | | | | | | | | | | | | | 0,2493 | | | | | | | |
| GENOTYPE x TIME | | | | | | | | | | | F (3, 105) = 0,8275 | | | | | | | | | | | | | 0,4816 | | | | | | | |
| GENOTYPE x SEX | | | | | | | | | | | F (3, 105) = 0,3006 | | | | | | | | | | | | | 0,8249 | | | | | | | |
| TIME x SEX | | | | | | | | | | | F (1, 105) = 0,0006780 | | | | | | | | | | | | | 0,9793 | | | | | | | |
| GENOTYPE x TIME x SEX | | | | | | | | | | | F (3, 105) = 1,364 | | | | | | | | | | | | | 0,2580 | | | | | | | |
|  | | | | | | | | | | | | | | | | | | | | | | | | | | | | | | | |
| **Shapiro-Wilk test** | | **WT-2M** | | | **MFS-2M** | | | **CD-2M** | | | | | | **CDMFS-2M** | | | | **WT-6M** | | | | | **MFS-6M** | | | | **CD-6M** | | | **CDMFS-6M** | |
| W | | 0,9805 | | | 0,9375 | | | 0,9502 | | | | | | 0,9283 | | | | 0,8976 | | | | | 0,9265 | | | | 0,9679 | | | 0,9387 | |
| **P value** | | 0,9325 | | | 0,3191 | | | 0,4281 | | | | | | 0,4314 | | | | 0,0735 | | | | | 0,2417 | | | | 0,8470 | | | 0,5051 | |
| **EF-2M** | | | | | | | | | | | | | | | | | | | | | | | | | | | | | | | |
| **ANOVA table** | | | | | | | | | **F (DFn, DFd)** | | | | | | | | | | | | **P value** | | | | | | | | | | |
| Genotypes | | | | | | | | | F (3, 60) = 3,910 | | | | | | | | | | | | P=0,0129 | | | | | | | | | | |
| **Dunnett's multiple comparisons test** | | | | | | | | | | | | | | | | **Adjusted P Value** | | | | | | | | | | | | | | | |
| WT vs. MFS | | | | | | | | | | | | | | | | 0,0729 | | | | | | | | | | | | | | | |
| WT vs. CD | | | | | | | | | | | | | | | | 0,0084 | | | | | | | | | | | | | | | |
| WT vs. CDMFS | | | | | | | | | | | | | | | | 0,0497 | | | | | | | | | | | | | | | |
|  | | | | | | | | | | | | | | | | | | | | | | | | | | | | | | | |
| **EF-6M** | | | | | | | | | | | | | | | | | | | | | | | | | | | | | | | |
| **ANOVA table** | | | | | | | | | **F (DFn, DFd)** | | | | | | | | | | | | **P value** | | | | | | | | | | |
| Genotypes | | | | | | | | | F (3, 52) = 8,714 | | | | | | | | | | | | P<0,0001 | | | | | | | | | | |
| **Dunnett's multiple comparisons test** | | | | | | | | | | | | | | | | **Adjusted P Value** | | | | | | | | | | | | | | | |
| WT vs. MFS | | | | | | | | | | | | | | | | 0,0007 | | | | | | | | | | | | | | | |
| WT vs. CD | | | | | | | | | | | | | | | | 0,0001 | | | | | | | | | | | | | | | |
| WT vs. CDMFS | | | | | | | | | | | | | | | | 0,0026 | | | | | | | | | | | | | | | |
|  | | | | | | | | | | | | | | | | | | | | | | | | | | | | | | | |
| **PROGRESSION** | | | | | | | | | | | | | | | | | | | | | | | | | | | | | | | |
| **Mixed-effects model (REML)** | | | | | | | | | | **F (DFn, DFd)** | | | | | | | | | | | | **P value** | | | | | | | | | |
| Time | | | | | | | | | | F (1, 113) = 12,40 | | | | | | | | | | | | 0,0006 | | | | | | | | | |
| Genotype | | | | | | | | | | F (3, 113) = 17,64 | | | | | | | | | | | | <0,0001 | | | | | | | | | |
| Time x Genotype | | | | | | | | | | F (3, 113) = 0,7211 | | | | | | | | | | | | 0,5414 | | | | | | | | | |
|  | | | | | | | | | | | | | | | | | | | | | | | | | | | | | | | |

**TABLE S6: CARDIAC REDOX STRESS**

| **OXPHOS COMPLEXES** | | | | | | | | | | | | | |
| --- | --- | --- | --- | --- | --- | --- | --- | --- | --- | --- | --- | --- | --- |
| **Kruskal-Wallis test** | | | | | | | | | | | | | |
| Kruskal-Wallis statistic | | | | | | | 21.37 | | | | | | |
| Number of groups | | | | | | | 8 | | | | | | |
| P value | | | | | | | 0,0033 | | | | | | |
| **Dunn's multiple comparisons test** | | | | | | | **Adjusted P Value** | | | | | | |
| WT-Males vs. WT-Female | | | | | | | >0,9999 | | | | | | |
| MFS-Male vs. MFS-Female | | | | | | | >0,9999 | | | | | | |
| CD-Male vs. CD-Female | | | | | | | >0,9999 | | | | | | |
| CDMFS-Male vs. CDMF-Female | | | | | | | >0,9999 | | | | | | |
|  | | | | | | | | | | | | | |
| **Shapiro-Wilk test** | | **WT** | | | **MFS** | | | | **CD** | | | **CDMFS** | |
| **W** | | 0,9245 | | | 0,9345 | | | | 0,9473 | | | 0,8767 | |
| **P value** | | 0,4304 | | | 0,4932 | | | | 0,6838 | | | 0,3247 | |
| **Kruskal-Wallis test** | | | | | | | | | | | | | |
| Kruskal-Wallis statistic | | | | | | | 20,97 | | | | | | |
| Number of groups | | | | | | | 4 | | | | | | |
| P Value | | | | | | | 0.0001 | | | | | | |
| **Dunn's multiple comparisons test** | | | | | | | **Adjusted P Value** | | | | | | |
| WT vs. MFS | | | | | | | 0,6703 | | | | | | |
| WT vs. CD | | | | | | | <0,0001 | | | | | | |
| WT vs. CDMFS | | | | | | | 0,6097 | | | | | | |
| MFS vs. CD | | | | | | | 0,0123 | | | | | | |
| MFS vs. CDMFS | | | | | | | >0,9999 | | | | | | |
| CD vs. CDMFS | | | | | | | 0,2892 | | | | | | |
|  | | | | | | | | | | | | | |
| **DHE LEVELS** | | | | | | | | | | | | | |
| **Shapiro-Wilk test** | **WT** | | **MFS** | **CD** | | **CDMFS** | | **WT** | | **MFS** | **CD** | | **CDMFS** |
| **W** | 0,9278 | | 0,9568 | 0,9606 | | 0,8999 | | 0,9234 | | 0,9273 | 0,9444 | | 0,9106 |
| **P value** | 0,5326 | | 0,7947 | 0,8240 | | 0,3302 | | 0,5524 | | 0,5282 | 0,6975 | | 0,4002 |
| **Two-WAY ANOVA SEX/GENOTYPE** | | | | | | | | | | | | | |
| **ANOVA table** | | | | **F (DFn, DFd)** | | | | | | **P value** | | | |
| Interaction | | | | F (3, 41) = 0,8410 | | | | | | P=0,4793 | | | |
| Sex | | | | F (1, 41) = 0,06844 | | | | | | P=0,7949 | | | |
| Genotype | | | | F (3, 41) = 11,83 | | | | | | P<0,0001 | | | |
|  | | | | | | | | | | | | | |
| **Shapiro-Wilk test** | | **WT** | | | **MFS** | | | | **CD** | | | **CDMFS** | |
| **W** | | 0,9118 | | | 0,9333 | | | | 0,9636 | | | 0,9497 | |
| **P value** | | 0,2253 | | | 0,3763 | | | | 0,8155 | | | 0,5940 | |
| **ORDINARY ONE -WAY ANOVA** | | | | | | | | | | | | | |
| **ANOVA table** | | | | **F (DFn, DFd)** | | | | | | **P value** | | | |
| Genotype | | | | F (3, 45) = 12,56 | | | | | | P<0,0001 | | | |
| **Dunnett's multiple comparisons test** | | | | | | | **Adjusted P Value** | | | | | | |
| WT- vs. MFS | | | | | | | >0,9999 | | | | | | |
| WT vs. CD | | | | | | | 0,0001 | | | | | | |
| WT vs. CDMFS | | | | | | | 0,6024 | | | | | | |
|  | | | | | | | | | | | | | |

**TABLE S7: CARDIAC MMP2**

| **MMP2 Expression** | | | | | |
| --- | --- | --- | --- | --- | --- |
| **Kruskal-Wallis test** | | | | | |
| Kruskal-Wallis statistic | | | 23.84 | | |
| Number of groups | | | 8 | | |
| P value | | | 0.0012 | | |
| **Dunn's multiple comparisons test** | | | **Adjusted P Value** | | |
| WT-Males vs. WT-Female | | | >0,9999 | | |
| MFS-Male vs. MFS-Female | | | >0,9999 | | |
| CD-Male vs. CD-Female | | | >0,9999 | | |
| CDMFS-Male vs. CDMF-Female | | | >0,9999 | | |
|  | | | | | |
| **Shapiro-Wilk test** | **WT** | **MFS** | | **CD** | **CDMFS** |
| **W** | 0,9340 | 0,8873 | | 0,9278 | 0,7829 |
| **P value** | 0,4523 | 0,1871 | | 0,4604 | 0,0583 |
| **Kruskal-Wallis test** | | | | | |
| Kruskal-Wallis statistic | | | 23,43 | | |
| Number of groups | | | 4 | | |
| P Value | | | <0.0001 | | |
| **Dunn's multiple comparisons test** | | | **Adjusted P Value** | | |
| WT vs. MFS | | | 0,0147 | | |
| WT vs. CD | | | 0,7797 | | |
| WT vs. CDMFS | | | 0,1848 | | |
| MFS vs. CD | | | <0,0001 | | |
| MFS vs. CDMFS | | | >0,9999 | | |
| CD vs. CDMFS | | | 0,0056 | | |
